# Multidimensional outcome of first-episode psychosis: a network analysis

**DOI:** 10.1017/S0033291724003465

**Published:** 2025-02-06

**Authors:** Manuel J Cuesta, Gustavo J Gil-Berrozpe, Ana M Sánchez-Torres, Lucía Moreno-Izco, Elena García de Jalón, Víctor Peralta, SEGPEPs Group

**Affiliations:** 1 Department of Psychiatry, Hospital Universitario de Navarra, Pamplona, Spain; 2 Navarra Institute for Health Research (IdiSNA), Pamplona, Spain; 3 Departament of Health Sciences, Universidad Pública de Navarra (UPNA), Pamplona, Spain; 4 Mental Health Department, Servicio Navarro de Salud, Pamplona, Spain

**Keywords:** First-episode psychosis, schizophrenia, network analysis, outcome measures, recovery

## Abstract

**Background:**

Few studies have examined the long-term outcomes of first-episode psychosis (FEP) among patients beyond symptomatic and functional remission. This study aimed to broaden the scope of outcome indicators by examining the relationships between 12 outcomes of FEP patients at 20.9 years after their initial diagnosis.

**Methods:**

At follow-up, 220 out of 550 original patients underwent a new assessment. Twelve outcomes were assessed via semistructured interviews and complementary scales: symptom severity, functional impairment, personal recovery, social disadvantage, physical health, number of suicide attempts, number of episodes, current drug use, dose-years of antipsychotics (DYAps), cognitive impairment, motor abnormalities, and DSM-5 final diagnosis. The relationships between these outcome measures were investigated using Spearman’s correlation analysis and exploratory factor analysis, while the specific connections between outcomes were ascertained using network analysis.

**Results:**

The outcomes were significantly correlated; specifically, symptom severity, functioning, and personal recovery showed the strongest correlations. Exploratory factor analysis of the 12 outcomes revealed two factors, with 11 of the 12 outcomes loading on the first factor. Network analysis revealed that symptom severity, functioning, social disadvantage, diagnosis, cognitive impairment, DYAps, and number of episodes were the most interconnected outcomes.

**Conclusion:**

Network analysis provided new insights into the heterogeneity between outcomes among patients with FEP. By considering outcomes beyond symptom severity, the rich net of interconnections elucidated herein can facilitate the development of interventions that target potentially modifiable outcomes and generalize their impact on the most interconnected outcomes.

## Introduction

First-episode psychosis (FEP) can lead to a wide range of illness trajectories that may be determined by premorbid conditions, concurrent intervening factors, access to specialized care, and adherence to treatment (Cuesta, 2023). FEP is more of a general descriptive term rather than a specific, and a relapsing–remitting pattern is the typical outcome in most FEP patients (Peralta et al., 2022; Tramazzo et al., 2024).

In many cases, the onset of FEP is insidious; up to 74% of FEP patients have a prodromic phase (Benrimoh et al., 2024). However, a small subset of them with a brief psychotic disorder diagnosis can have an acute onset within hours or a few weeks (Ajnakina et al., 2017; Fusar-Poli et al., 2022). Strategies to reduce the time to intervention in FEP patients are critical since the duration of untreated illness (DUP) is strongly related to more severe symptoms and a lower likelihood of remission at follow-up (Howes et al., 2021). Once the illness began, longitudinal studies revealed that deterioration can occurs within 3–5 years of FEP diagnosis, a period of time known as “the critical period,” and may endure over time(Hansen et al., 2023; O’Keeffe et al., 2019; Peralta et al., 2022; Starzer et al., 2023).

Clinical remission of symptoms has been the most commonly outcome of FEP, although there is growing evidence to support research on psychosocial functioning and personal recovery measures to better account for the heterogeneity of FEP outcomes (Peralta et al., 2022). Personal recovery is a relatively new domain usually neglected in outcome studies and it showed only small to medium significant associations with clinical recovery (Van Eck et al., 2018a). However, the inclusion of the patient’s perspective not only may enable a better capture of the own process of recovery (Felix et al., 2024) but it may also help in understanding the heterogeneity of outcomes of FEP patients (Griffiths et al., 2022; Shanks et al., 2013).

At least eight additional intervening processes may also influence the course of FEP patients. First, a continued cannabis use after the onset of psychosis may favor adverse outcomes, such as increased relapse rates, lengthier hospital stays, and more severe positive symptoms (Schoeler et al., 2016). Second, the number of episodes is a strong predictor factor of poor outcome (Solmi et al., 2023). Third, cumulative doses of antipsychotic drugs showed evidence of associations with a generalized decrease in grey matter volume (Fusar-Poli et al., 2013; Haijma et al., 2013). Fourth, the close relationship between mental and physical health has a significant impact on FEP patients’ outcomes over time. FEP patients in psychosis may resist seeking medical attention, which raises their risk of physical comorbidities (Correll et al., 2022). Fifth, FEP patients are highly vulnerable to social exclusion, which increase their poor prognosis and drift to other forms of disadvantage (Peralta et al., 2024). Sixth, suicide trajectories have relevance for prognosis since persistence or worsening of suicidal ideation and behaviors during follow-up may led to poor outcomes in FEP patients (Gohar et al., 2023). Seventh, cognitive impairment is a central feature of psychosis and the magnitude of the deficit is associated with poor outcome measures, such as negative symptoms, social and vocational outcomes and loss of gainful employment (Cuesta et al., 2018; Ferruccio et al., 2021; Peralta & Cuesta, 2017a). Eighth, despite neuromotor domain has been scarcely studied, there is strong evidence supporting that baseline neurological soft-signs predict poor functional or symptomatic outcome in FEP patients (Cuesta et al., 2018; Peralta & Cuesta, 2017b).

Taken together, the outcomes of FEP can be better understood from a dynamic, multidimensional process whereby multiple risk factors and protective factors interact over time (Power, 2017) and widening the scope of research to include other outcome measures may enhance personalized care in FEP patients (Cuesta et al., 2022).

Thus, the next step in this line of research is to examine the interrelationships of 12 outcome measures at the long-term follow-up of FEP patients and to analyze the interdependence of outcomes using network analysis. Network analysis does not assume underlying latent causes (Borsboom et al., 2021) among outcome measures, such as factor analysis. Instead, network analysis computes and displays a representation of potential causal links between outcomes that lead to their co-occurrence (Christensen & Golino, 2021).

The 12 outcome measures were as follows: symptom severity, functioning, personal recovery measures, social disadvantage, physical disability, drug abuse status, the number of suicide attempts, the number of episodes, the lifetime antipsychotic exposure, a final DSM-5 diagnosis, a global cognitive score, and a motor score. Additionally, the interconnections of every relevant outcome measure were examined to determine their net interconnections.

## Methods

### Sample

The participants were recruited drawn from the SEGPEPS study (Estudio de seguimiento de Primeros Episodios de Psicosis de Navarra), which was a longitudinal cohort study of consecutively admitted patients with FEP between 1990 and 2008. This cohort was prospectively reassessed across multiple domains (the data were collected from 2018 to 2021). A total of 243 of the 510 patients who were evaluated at baseline (46.4% of the initial sample and 57.3% of the survivors) were successfully followed up and were thus included in the study.

The inclusion criteria were as follows: a) a diagnosis of FEP in accordance with either the DSM-III-R or DSM-IV criteria; b) aged between 15 and 65 years; c) living in the hospital’s catchment area; d) completing a 6-month assessment after discharge; e) availability of close relatives to provide general background information; and f) signed a written informed consent. The exclusion criteria were as follows: a) a history of major medical or neurological conditions, b) a suspected or confirmed diagnosis of drug-induced psychosis, and c) an IQ less than 70, which indicated an intellectual disability. A full description of the SEGPEPs study has been described elsewhere (Peralta et al., 2021).

Written informed consent was provided by each participant, and if applicable, their legal representatives. Ethical approval was obtained from the local ethical committee. The authors declare that all the methods used in this work complied with the ethical requirements of the institutional and national committees on human experimentation.

### Assessment methodology and raters

Participants were evaluated by the senior authors (VP or MJC) at the time of FEP. Two trained psychiatrists who are highly skilled at assessing psychosis (LMI and EGJ) conducted direct interviews with patients as well as a close informant or a relative and carried out patient assessments. We attempted to track down the participants following a three step-strategy. First, phone and postal mail to patients. Second, two months after the initial contact attempt, those who did not reply were approached either directly or through their general practitioner or treating psychiatrist. Third, we accessed to the General Register Office and electronic health information to find deceased patients. The interviewers were blinded to the participant’s baseline data. At follow-up, patients’ diagnoses were updated based on the DSM-5 criteria^15^ after considering all the information collected via the Comprehensive Assessment of Symptoms and History (CASH) (Andreasen et al., 1992) and the information obtained from specific assessment instruments to account for relevant variables not included in the CASH.

A Life Chart Schedule (LCS) for each subject that includes lifetime symptoms, functioning scores, and other illness-related variables, such as medication history, medical and psychiatric comorbidities, drug abuse, significant life events, and service use over the course of the illness, was drawn from the Past and Lifetime History sections of the CASH. Clinical records from the Navarra health service’s computerized database, which houses the registry of all public medical and mental health services, were used as the additional sources of information.

### Multidimensional assessment of outcomes

Twelve outcomes were assessed at the end of the follow-up period. These outcomes were examined separately, but some overlap was acceptable. Briefly, symptom severity was evaluated by adding the total positive, negative, depression, mania, and catatonia global rating scores of the CASH. Functional recovery was evaluated using the total score of the Social and Occupational Functioning Assessment Scale (SOFAS) from the past year (Goldman et al., 1992, p. 201). Personal recovery was evaluated using the total score of the Questionnaire on the Process of Recovery (QPR-15). The QPR has good psychometric properties (reliability and validity), and their scorings showed substantial correlation with relevant personal aspects of FEP patients in the recovery process, such as empowerment, quality of life, and overall psychological well-being (Law et al., 2014; Leendertse et al., 2018; Shanks et al., 2013). Social disadvantage (SocDis) was evaluated using a composite score that included the sum of the following dichotomous variables: single status, living with one’s own family, owner of one’s own home, skilled or specialized profession, paid work, and documented psychiatric disability. Physical disability (PhyDis) was evaluated using a composite score that included the sum of the following dichotomized variables: metabolic syndrome criteria (Sm et al., 2005), more than one associated medical illness, and the EQ-5D subjective thermometer-like visual analogue scale (a cut-off score of ≥50 on the Euroquol 5D) (Herdman et al., 2011).

The drug abuse status outcome was assessed at the end of follow-up using the Addiction Severity Index (McLellan et al., 1992), which assesses all drugs except alcohol and tobacco. The number of suicide attempts (SuicAtt) and the number of episodes (Episod) were recorded throughout the follow-up by calculating the means of the LCS. Lifetime antipsychotic exposure was measured using the dose-years of antipsychotics (DYAps), which is defined as the product of the dose and the time on that dose (in years) (Andreasen et al., 2010). Each patient received a score of belonging based on the final DSM-5 diagnosis, which was clustered into three groups: schizophrenia or schizoaffective disorder (score of 3), bipolar or affective disorder (score of 2), and other psychoses (score of 1).

The total score on the Screen for Cognitive Impairment in Psychosis (SCIP) (Pino et al., 2008) was examined as a cognitive outcome. The motor score was obtained from the sum of dichotomized scores on parkinsonism (Simpson & Angus, 1970), akathisia (Barnes, 1989), dyskinesia (Guy et al., 1986), and the total catatonia score on the CASH. The total scores on the four scales were subjected to a median split.

### Statistical analyses

We first conducted univariate analysis to examine differences in sociodemographic and psychopathological variables between patients who completed follow-up and those who did not complete follow-up (χ2 or ANOVA tests).

We used Spearman correlations and exploratory factor analysis with Oblimin rotation to gain insights into the interactions and latent constructs of outcome measures. A heatmap of correlations was constructed, and the Bonferroni correction was applied (0.05/66; *r* = 0.024, *p* ≤ 0.0007).

Given that our main aim was the examination of the direct interrelationships between every pair of outcomes a network analysis was carried out. Network analysis is a powerful statistical methodology for examining the interrelationships between complex systems in psychiatric research (Borsboom, 2017). This approach focuses on the dynamic nature of mental diseases as systems and allows for inferences regarding the underlying mechanisms of psychopathology and other domains in psychosis (Galderisi et al., 2020; Gil-Berrozpe et al., 2023; Peralta et al., 2020). Regularized partial correlation (using group least absolute shrinkage and selection operator (gLASSO) with extended Bayesian information criteria (EBIC) model selection was used to estimate the network architecture. A weighted network structure can be used to visualize partial correlations. Each node in the network represents an outcome, and each edge indicates the relationship between two outcomes once all other variables have been controlled. The partial correlation coefficients are the edge weights.

Centrality indices (betweenness, closeness, strength, and expected influence) were computed to measure the significance of every node in the network. The strength is determined by summing all the edge weights that are directly connected to a node. The betweenness is determined based on the number of times a node appears on the shortest path linking the two other nodes. Closeness is the inverse of the weighted sum of the distances from all the other nodes in the network. It measures the ease with which a given node can connect with every other node. The strength of a given node is the sum of the weights of its connections. The expected influence measure is conceptually equivalent to strength, although it accounts for the real value (positive or negative) of edges. To ensure the validity of the findings, edge and centrality stability were assessed using nonparametric and case-dropping bootstrapping approaches (Borsboom et al., 2021; Epskamp et al., 2017). In addition, to quantify the stability of the centrality estimates, we employed the correlation stability coefficient (CS coefficient). Coefficients greater than 0.25 are recommended, but it is preferred for them to be greater than 0.50.

All the statistical analyses were conducted using R statistical software by means of qgraph, Bootnet (R Core Team 2016), and JASP statistical software (JASP v 0.18.1.0).

### Results

The baseline FEP cohort comprised 510 patients, and 243 (47.6%) completed the follow-up assessments. The primary clinical and demographic factors did not differ between the followed-up and non-followed-up participants, except for the mean age, which was significantly younger in the follow-up sample (Peralta et al., 2022). The final sample for this study consisted of 220 patients because 23 patients did not undergo cognitive examination (SCIP) (Table 1).

**Table 1.** Sociodemographic, clinical, diagnostic, and neurocognitive characteristics of the long-term follow-up of first-episode patients (*N* = 220)

	Mean	SD
Age, y	47.9	10.2
Gender, *n* (male/female)	123/97	
Single status, *n* (yes/no)	152/68	
Socioeconomic status score (1–5)	3.02	0.7
Education, years	11.4	3.3
Follow-up, y	20.6	5.4
Diagnosis, *n* (%):		
Schizophrenia	99 (45.0)	
Schizophreniform disorder	6 (2.7)	
Brief psychotic disorder	17 (7.7)	
Delusional disorder	3 1.4)	
Schizoaffective disorder	38 (17.3)	
Mania/bipolar disorder	39 (17.7)	
Major depressive disorder	9 (4.1)	
Psychotic disorder NOS	9 (4.1)	
Outcome measures*		
Symptom severity	9.59	8.1
Functioning	63.89	20.7
Personal recovery	43.13	11.0
Social disadvantage	1.90	1.6
Physical disadvantage	0.86	0.9
Drugs abuse	1.35	1.83
Number of episodes	7.69	7.9
Number of suicide attempts	1.09	2.8
Dose-Years antipsychotics	53.37	43.6
DSM 5 diagnosis (3 groups)	1.76	0.7
SCIP total score	59.86	20.6
Neuromotor total score	1.36	0.7

*Note*: SCIP, cognitive score (the screen for cognitive impairment in psychiatry)

* = See Methods for a description of the composition of the outcome measures.

### Spearman correlations

An examination of the matrix of correlations revealed that most outcome measures were strongly associated with one another, and most of the correlations remained significant after the Bonferroni correction (Table 2). Symptom-related and functioning measures were significantly associated with the other outcome measures, except for Abuse. Personal recovery was significantly associated with 10 out of the 12 outcome measures, and SocDis was significantly associated with all but three of the outcome measures (Abuse, Episod, and SuicAtt). PhyDis was not associated with SocDis, abuse, or Episod. Abuse was not significantly associated with any outcome measures. Episod was significantly associated with symptoms, functioning-related SocDis, SuicAtt, DYAps, and SCIP. SuicAtt was associated with symptom severity, functioning-related personal recovery, and Episod. DYAps was associated with all measures except abuse. The final DSM 5 diagnosis was associated with symptom severity, functioning, personal recovery, SocDis, DYAps and SCIP. SCIP was associated with all outcomes except Episod and SuicAtt. The motor score was associated with all outcome measures except for PhyDis, abuse, Episod, number of suicide attempts, and SCIP (Table 2).

**Table 2.**
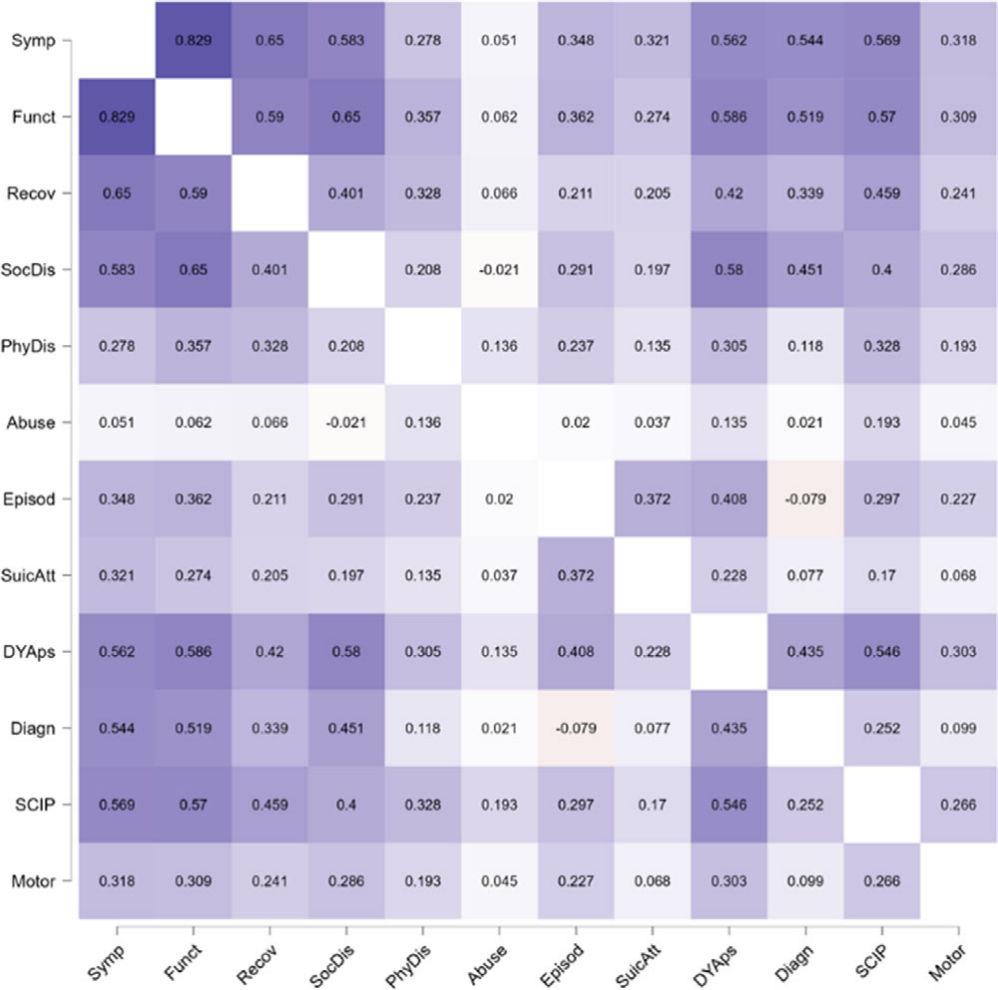
Heatmap of Spearman’s coefficients correlations between long-term outcome measures of FEP patients

* =Bonferroni correction (*r* = 0.024. *p* ≤ 0.0007)

Notes: Symp, Symptomatic severity; Funct, Functioning; Recov, personal recovery; SocDis, social disadvantage; PhyDis, physical disadvantage; Abuse, drugs abuse; SuicAtt, number of suicide attempts; Episod, number of episodes; DYAps, dose-years of antipsychotics drugs; Diagn, final DSM 5 diagnosis (categorized as follows: 1 = Other Psychosis; 2 = Bipolar and affective psychosis; 3 = Schizophrenia and Schizoaffective disorders); SCIP, SCIP total score: Motor: Sum of the scores of motor scales

### Exploratory factor analysis of the 12 outcomes

Exploratory factor analysis was conducted using a primary axis and oblique rotation (Oblimin) to identify the latent variable structure and investigate the relationships between the outcomes. Visual examination of the scree plot revealed that a two-factor solution (eigenvalue≥1.3) best fit the observed data. This solution explains 42.8% of the variance. Eleven of the 12 outcomes loaded on the first factor; Episod was the one outcome that loaded onto the second factor (Table 3).

**Table 3.** Exploratory factor analysis of the 12 long-term outcome measures of FEP patients (Oblimin rotation): Factor characteristics and rotated matrix structure

Factor characteristics							
	Eigenvalues	SumSq. loadings	Unrotated solution Proportion var.	Cumulative	SumSq. loadings	Rotated solution proportion var.	Cumulative
Factor 1	4.570	4.125	0.344	0.344	3.910	0.326	0.326
Factor 2	1.322	1.010	0.084	0.428	1.223	0.102	0.428

Factor loadings (structure matrix)		
	Factor 1	Factor 2
Symp	0.867	0.278
Funct	0.905	0.319
Recov	0.685	
SocDis	0.667	0.273
PhyDis	0.385	0.210
Abuse		
Episod	0.329	0.999
SuicAtt		0.343
DYAps	0.637	0.286
Diagn	0.526	
SCIP	0.675	0.220
Motor	0.370	0.227

*Notes*: Symp, Symptomatic severity; Funct, Functioning; Recov, personal recovery; SocDis, social disadvantage; PhyDis, physical disadvantage; Abuse, drugs abuse; SuicAtt, number of suicide attempts; Episod, number of episodes; DYAps, dose-years of antipsychotics drugs; Diagn, final DSM 5 diagnosis (categorized as follows: 1 = Other Psychosis; 2 = Bipolar and affective psychosis; 3 = Schizophrenia and Schizoaffective disorders); SCIP, SCIP total score: Motor: Sum of the scores of motor scales

### Network analysis

**Figure 1.**
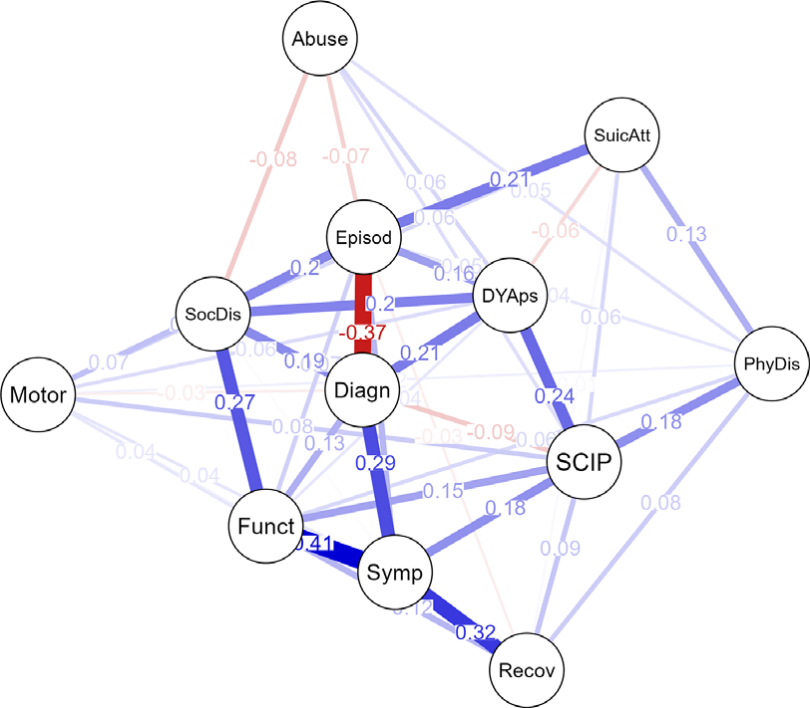
Network analysis of the 10 measures of outcome at the long-term follow-up of FEP patients. *Note*: Symp, symptom severity; Funct, functioning (SOFAS total score of the last year); Recov, QPR-15 total score; SocDis, social disadvantage score; PhyDis, physical disadvantage score; Abuse, ASI (Addiction Severity Index) total score last year; Episod, number of episodes; SuicAtt, number of suicide attempts; DYAps, dose-years of antipsychotic drugs; Diagn, DSM 5 final diagnosis (three groups); SCIP, cognitive score (the screen for cognitive impairment in psychiatry; Motor, motor abnormalities.

**Figure 2.**
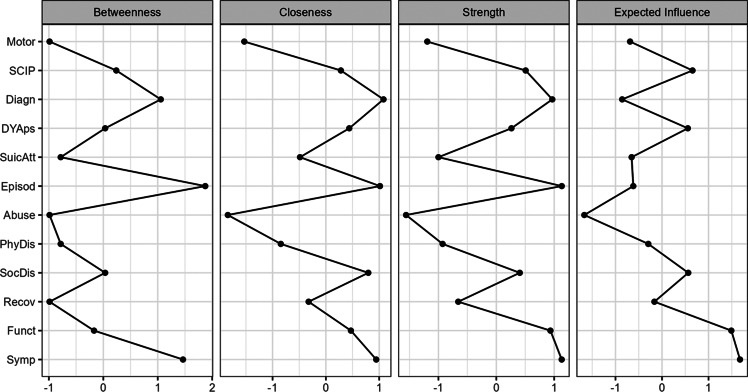
Centrality indexes of the network. *Note*: Symp, symptom severity; Funct, Functioning (SOFAS total score of the last year); Recov, QPR-15 total score; SocDis, social disadvantage score: PhyDis, physical disadvantage score; Abuse, ASI (Addiction Severity Index) total score last year; Episod, number of episodes; SuicAtt, number of suicide attempts; DYAps, dose-years of antipsychotic drugs; Diagn, DSM 5 final diagnosis (three groups); SCIP, cognitive score (the screen for cognitive impairment in psychiatry; Motor, motor abnormalities.

The network structure is shown in Figure 1. Network analysis revealed a high degree of interconnectedness among the 12 outcome measures without isolated nodes. The regularized network retained 69.6% of all possible edges (46/66). The CS coefficient of strength was 0.67, suggesting the stability of the network and that the centrality indices can be reliably interpreted (Figure 2). Edge-weight accuracy indices with confidence intervals overlap, thus suggesting that they are not significantly different from one another. However, betweenness tends to be less stable than closeness and strength, as the sample size was decreasing at random to retain, with 95% certainty, a correlation coefficient of at least 0.7 between the sample’s centrality indices and case-dropped bootstraps’ centrality indices (Epskamp et al., 2018) (Supplemental Figure 1).

The most significant nodes in the network graph with respect to their strength and expected influence were symptom severity, functioning, SocDis, diagnosis, SCIP, DYAps, and number of episodes. Nonetheless, visual inspection of the network revealed a rich network of interconnections between these main outcome measures and the others (Figure 1).

The highest degree of interconnectedness was observed for symptom severity regarding functioning and personal recovery (0.413 and 0.323, respectively) (see Figure 1 and Supplementary Table 1 for the weight matrix between outcome measures). Moreover, symptomatic recovery and functioning showed a high degree of interconnectedness with diagnosis and SCIP scores (0.291 and 0.179 direct associations, respectively) but a lesser degree of interconnectedness with Episod, SocDis, and motor score. Personal recovery showed a high degree of interconnectedness with symptomatic and functioning outcomes (0.323 and 0.118, respectively) (Supplementary Table 1).

Episod showed a strong inverse connection with diagnosis in the network graph, which indicates that non-schizophrenia patients have a high number of episodes (0.116), since higher scores at diagnosis reflect schizophrenia diagnosis and lower scores on affective and other psychoses.

The final DSM 5 diagnosis showed strong relationships with symptomatic recovery, duration of antipsychotic drug use, SocDis, and functioning and strong and inverse relationships with Episod. The cognitive score (SCIP total score) revealed strong associations with DYAps, PhyDis, symptomatic recovery, and functioning.

DYAps also showed direct associations with the final diagnosis and SCIP in addition to the associations with outcomes of SocDis and Episod.

SocDis was directly associated with DYAps and Episod. PhyDis was positively associated with SCIP and SuicAtt. Drug abuse was weakly and directly associated with DYAps, SCIP, and PhyDis and inversely associated with Episod and SocDis. Episod showed a strong inverse relationship with the final diagnosis and a direct association with SuicAtt. SuicAtt showed strong associations with Episod and PhyDis and a weaker relationship with SCIP. In addition, the motor node was interrelated with Episod, SocDis, SCIP, DYAps, symptom severity, functioning, final diagnosis and PhyDis.

## Discussion

This study aimed to broaden the scope of measures for the assessments of the long-term outcome of FEP patients by examining the interrelationships between symptom severity, functioning, personal recovery outcomes, and nine other complementary outcome measures. The key findings were as follows. First, most of the outcomes examined herein were significantly intercorrelated, and symptom severity, functioning, and personal recovery showed the strongest correlations (Spearman correlation coefficients ≥0.59) with the other measures. Second, an exploratory factor analysis of the 12 outcomes revealed a two-factor model, with 11 of the 12 outcomes loaded onto one factor and the one remaining outcome (Episod) loaded onto the second factor. Third, network analysis showed that symptom severity, functioning, SocDis, diagnosis, SCIP, DYAps, and Episod were the most interconnected outcome measures. This finding enabled a better understanding of the heterogeneity of outcomes in FEP patients.

Although most of the outcome measures examined here showed strong relationships and high shared variance when analysed using correlation and factor analyses, only network analysis enabled the extraction of specific interconnections between nodes by partiallizing the influence of the remaining network nodes.

Symptom severity and functioning merged as central nodes of the network because of their strong interconnectedness, although the network analysis displayed a rich net of specific links between the 12 outcome measures.

Functioning showed strong association with cognitive impairment but this association was lower than with symptom severity. This result could be at first instance unexpected because there is consistent evidence that reality distortion dimension and disorganization dimensions showed respectively weak and moderate associations with cognitive impairment (Dominguez et al., 2009; Galderisi et al., 2009). However, our symptom severity score was made up of all psychopathological dimensions including negative symptoms that have strong associations with cognitive impairment in FEP patients (Zhang et al., 2024).

Symptom severity and functioning showed consistent but weaker connections with personal recovery, thus providing evidence that personal recovery is conceptually different but maintains strong links to these variables (Leamy et al., 2011; Morgan et al., 2021; O’Keeffe et al., 2019; Van Eck et al., 2018b). Even in cases of severe psychotic symptoms, patients frequently report positive personal recovery based on their individual experience (Anthony, 1993; Van Eck et al., 2018b). This discrepancy between self-reports and behavioral assessments may be due to the distinct response processes that are often non-coincident in a single construct (Dang et al., 2020). Despite an increasing amount of evidence suggesting that personal recovery is a crucial outcome for FEP patients (Leamy et al., 2011; Morgan et al., 2021; O’Keeffe et al., 2019; Van Eck et al., 2018b), it has been neglected in pharmacological studies (Béchard et al., 2024). In fact, the primary justification for FEP patients to continue antipsychotics is their effectiveness, whereas the main justification for quitting antipsychotics is their side effects (Ae et al., 2023). However, in only 19% of patients with schizophrenia, a decrease in psychotic symptoms is a good enough reason to continue taking antipsychotics when personal recovery is defined as the study’s primary goal (Leamy et al., 2011).

Impaired social functioning is a long-term core outcome in patients with schizophrenia and related disorders (Velthorst et al., 2017). Under the umbrella of the social domain, different terms, such as social functioning (Goldman et al., 1992, p. 201), primary and secondary negative symptoms (Galderisi et al., 2018; Giordano et al., 2022), social exclusion (Peralta et al., 2024), social disability and social disconnection (Green et al., 2018), and nonsocial and social cognition (Green et al., 2019), have been investigated. SocDis examined in our study was mainly based on economic and residential independence and employment status (Huxley et al., 2021; Warner, 1994, 2009), thus differentiating this variable from an individual’s level of social and occupational functioning (Goldman et al., 1992). Both domains were strongly connected in our network, but they also showed a differentiated pattern of connections, such as the link between SocDis and DYAps. This interconnection may substantiate findings in the literature reporting better functioning and work performance in patients taking low doses of antipsychotics (APs) or patients taking no APs (Harrow et al., 2017; Wunderink et al., 2009). In addition, it is very common that higher doses of APs are prescribed for treatment-resistant patients (Hegarty et al., 1994; Huxley et al., 2021; Jaaskelainen et al., 2014), who very often are prone to SocDis. Another interesting strong relationship observed in our network was the association between SocDis and Episod, which emphasizes the role of the social component as a risk factor for relapse; this relationship has been demonstrated for social withdrawal (Almuqrin et al., 2023).

We found strong interconnections between DYAps and cognitive impairment. Previous studies have reported beneficial improvements in cognitive functioning in patients with schizophrenia treated with second-generation APs (Bilder et al., 2002; Cuesta et al., 2009; Harvey & Bowie, 2012; Woodward et al., 2005). However, most of these improvements disappeared when the practice effect was controlled (Goldman et al., 1992). Indeed, there is cumulative evidence that their antagonism of the dopamine D_2_ receptor, which is essentially the main target in the acute phase and relapse prevention of schizophrenia, and the anticholinergic action of APs may induce cognitive impairment in the long-term (Ballesteros et al., 2018; Feber et al., 2023; Hulkko et al., 2017; Husa et al., 2017). Moreover, the higher affinity of APs for the α_1_ adrenergic, muscarinic M_1_, and histamine H_1_ receptors seems to be related to cognitive impairment (Baldez et al., 2021). On the other hand, superior cognitive outcomes have been reported in studies focusing on medically guided dose reduction of APs both in the short-term (Singh et al., 2022) and long-term(Harrow et al., 2017; Wunderink et al., 2009).

DYAps demonstrated strong connectedness with Episod within the network. This is likely because patients who experienced more episodes needed higher doses of APs, as non-adherence and resistance are frequently linked to the prescription of higher doses of antipsychotic medications, which in turn results in a high number of admissions (Rubio et al., 2021).

A high number of episodes showed a high degree of strength in our network, and it was strongly connected with SocDis, DYAS, SuicAtt, and diagnosis. Episod, which usually reflects relapses, may lead to readmissions and is a proxy measure of course severity (Owusu et al., 2022). Moreover, the degree of deterioration has been found to be significantly correlated with the number of relapses (Emsley et al., 2013). However, the link between Episod and SocDis is clearly suggestive of bidirectionality since both factors are poor indicators of prognosis and both factors are correlated with illness severity. Another finding in the network was a robust link between Episod and suicide attempts, which is in accordance with studies reporting that suicidal ideation or risk is one of the major reasons for readmission in psychosis patients (Mellesdal et al., 2010; Tedeschi et al., 2020).

Studies on medical comorbidities have reported high rates of metabolic syndrome and cardiovascular and respiratory diseases during the course of psychosis, which can undermine mental health recovery (Morgan et al., 2021; Suetani et al., 2021). Jester et al (Jeste, 2023, p. 202; Jester et al., 2023) suggested that cognitive impairment and physical comorbidity may be neurobiological pathways expressing the consequences of major social determinants of health. This suggestion can be visualized in our network in the form of the high connectedness between the two domains. Moreover, compared to the general population, people with schizophrenia experience a faster rate of physical ageing and mild cognitive impairment (Jeste et al., 2011).

There is consistent evidence that metabolic syndrome (MetS) is linked to cognitive impairments in the general population as well as in patients with schizophrenia (Bora et al., 2017). Indeed, it has been shown that MetS, diabetes and cardiovascular risk factors (hypertension) are significantly associated with global cognitive impairment in people with schizophrenia (Hagi et al., 2021).

There was a strong interrelationship between SuicAtt and Episod in our network, as both outcomes are commonly observed found during acute episodes. However, few studies have examined the relationship between suicide attempts and PhyDis in the context of psychosis. However, the results from the Adult Psychiatric Morbidity Survey 2007, a cross-sectional study in England, reported that physical multimorbidity is associated with significantly greater odds of suicidal ideation and suicide attempts (Smith et al., 2023).

The association between suicidality and cognitive performance was unclear in the literature because most of the relevant studies were cross-sectional. Some studies have shown an association between suicidality and preserved cognitive function in schizophrenia patients (De Hert et al., 2001; Delaney et al., 2012; Kim et al., 2003) or FEP patients (Kim et al., 2023), while others have not shown differences in neurocognitive performance between non-attempters and patients with multiple suicide attempts (Potkin et al., 2003). The interconnectedness between suicide attempts and cognitive impairment in our network was consistent with the results of a study carried out in the Spanish general population, which reported that worse cognitive functioning was associated with more frequent suicidal ideation; however, the association notably was stronger among patients with depression (Lara et al., 2015). In fact, for affective disorders, cognitive impairment was significantly associated with a higher lifetime rate of attempted suicide (Vocisano et al., 1997).

Some unexpected findings were observed in our network. For example, symptom severity and functioning were not related to drug abuse. Moreover, drug abuse showed robust relationships with higher cumulative doses of antipsychotics (DYAps) and cognitive impairment but lower SocDis and number of episodes. These striking findings may be explained by the sociodemographic characteristics of our sample, since the prevalence of drug users in our sample progressively decreased over time, and only 16.5% reported active drug abuse at the long-term follow-up. The low prevalence of active drug abuse could be consistent with the tendency to reduce cannabis abuse over time in the general population and in patients (Choi et al., 2016; Han & Palamar, 2020). In addition, the prevalence of drug abuse for the patients in the 45–54 years age group, which corresponds to the mean age of our sample (48.1 ± 10.7), was five times lower than that for the 15–24 years age group (Manthey et al., 2021).

High levels of motor abnormalities were consistently associated with cognitive impairment and SocDis and moderately associated with symptom severity, functioning, and DYAps. Our motor node was composed predominantly of extrapyramidal signs (parkinsonism, akathisia, and dyskinesia) since ratings of catatonia were very low. The link between motor abnormalities and cognitive impairment has been largely reported by our group and others, who demonstrated a significant association between parkinsonian signs and cognitive impairment (Cuesta et al., 2018, 2021; Fritze et al., 2022, 2024). Moreover, a recent systematic review concluded that motor abnormalities were related to symptomatic and functional deterioration (Pieters et al., 2022) and that motor abnormalities are highly correlated but also modulated with antipsychotic treatment from naïve status to clinical remission in FEP patients (Peralta & Cuesta, 2010). Finally, focusing on the association of motor abnormalities with SocDis, the latter may favour the emergence of negative symptoms (Zahid & Best, 2021) that are closely associated with parkinsonism in patients with schizophrenia (Cuesta et al., 2018; Nadesalingam et al., 2022).

The last outcome we examined was diagnosis, which showed strong links with most of the other outcome measures. Implicitly, many of the outcomes, especially the severity of psychotic symptoms and poor psychosocial functioning (American Psychiatric Association, 2013), contribute to a final DSM-5 diagnosis. Therefore, these interconnections of diagnosis are not immune to circularity but instead provide additional evidence regarding the contribution of other outcomes to the final diagnosis.

## Conclusion

The main strengths of the current study include its broad scope of long-term outcomes among FEP patients. Twelve outcomes were assessed herein, and network analysis was used to gain an understanding of the rich and varied associations between these outcomes and to account for the clinical heterogeneity of FEP patients. This is the first study to emphasize and analyse 12 long-term outcome measures among FEP patients.

This study also has clinical implications since most of the outcomes are modifiable factors. Hypothetically, the high degree of interconnectedness between outcome measures may explain how targeting the prevention or treatment of one specific outcome measure might lead to generalized beneficial effects on the other connected outcomes.

## Limitations

A potential limitation of this study is the inclusion of all psychosis subtypes; therefore, no direct inferences can be made about specific diagnoses. However, we used the final DSM-5 diagnosis as the outcome, which allowed us to assess the three groups of psychosis disorders.

This follow-up study included FEP patients who were admitted to the hospital. This group had a high rate of attrition even though it was lower than in other long-term follow-up studies of FEP patients, such as OPUs study (Hansen et al., 2023), RAISE-ETP trial (Robinson et al., 2022) and STRATA study (Homman et al., 2021). These two limitations limit the generalizability of the results to the entire population of patients with FEP. Moreover, as this was a long-term follow-up study over more than 20 years, it is likely that future outcomes of current FEP patients will be enhanced due to improvements in services.

The selection of outcome measures was focused on the most relevant measures identified from the literature and from clinical practice. However, there may be other relevant outcomes that were not included herein that will enrich future studies contributing to personalize the scope of outcomes in FEP patients.

## Supporting information

Cuesta et al. supplementary material Cuesta et al. supplementary material

## Supplementary material

The supplementary material for this article can be found at http://doi.org/10.1017/S0033291724003465.
