# Multidimensional outcome of first-episode psychosis: a network analysis – CORRIGENDUM

**DOI:** 10.1017/S0033291725101098

**Published:** 2025-08-01

**Authors:** Manuel J Cuesta, Gustavo J Gil-Berrozpe, Ana M Sánchez-Torres, Lucía Moreno-Izco, Elena García de Jalón, Víctor Peralta, SEGPEPs Group

**Affiliations:** 1 Department of Psychiatry, Hospital Universitario de Navarra, Pamplona, Spain; 2 Navarra Institute for Health Research (IdiSNA), Pamplona, Spain; 3 Departament of Health Sciences, Universidad Pública de Navarra (UPNA), Pamplona, Spain; 4 Mental Health Department, Servicio Navarro de Salud, Pamplona, Spain

When this article was originally published in Psychological the funding statement was incorrect and has been updated to the following:

This work was funded by the Carlos III Health Institute, the Spanish Ministry of Science, Innovation and Universities, the European Regional Development Fund (ERDF/FEDER) (PI 19/1698 and RD21/0009/0025) and the Government of Navarra’s Health Department (20/21)

The authors apologise for this error.
