# Supplementary material for: Multidimensional outcome of first-episode psychosis: a network analysis
Source: Psychol Med. 2025 Feb 6;55:e29. doi: 10.1017/S0033291724003465 (PMC12017362; doi:10.1017/S0033291724003465)
Supplement: Cuesta et al. supplementary material [file S0033291724003465sup001.docx]

**Supplemental figure 1: Edge-weight accuracy indexes with confidence intervals of the network**


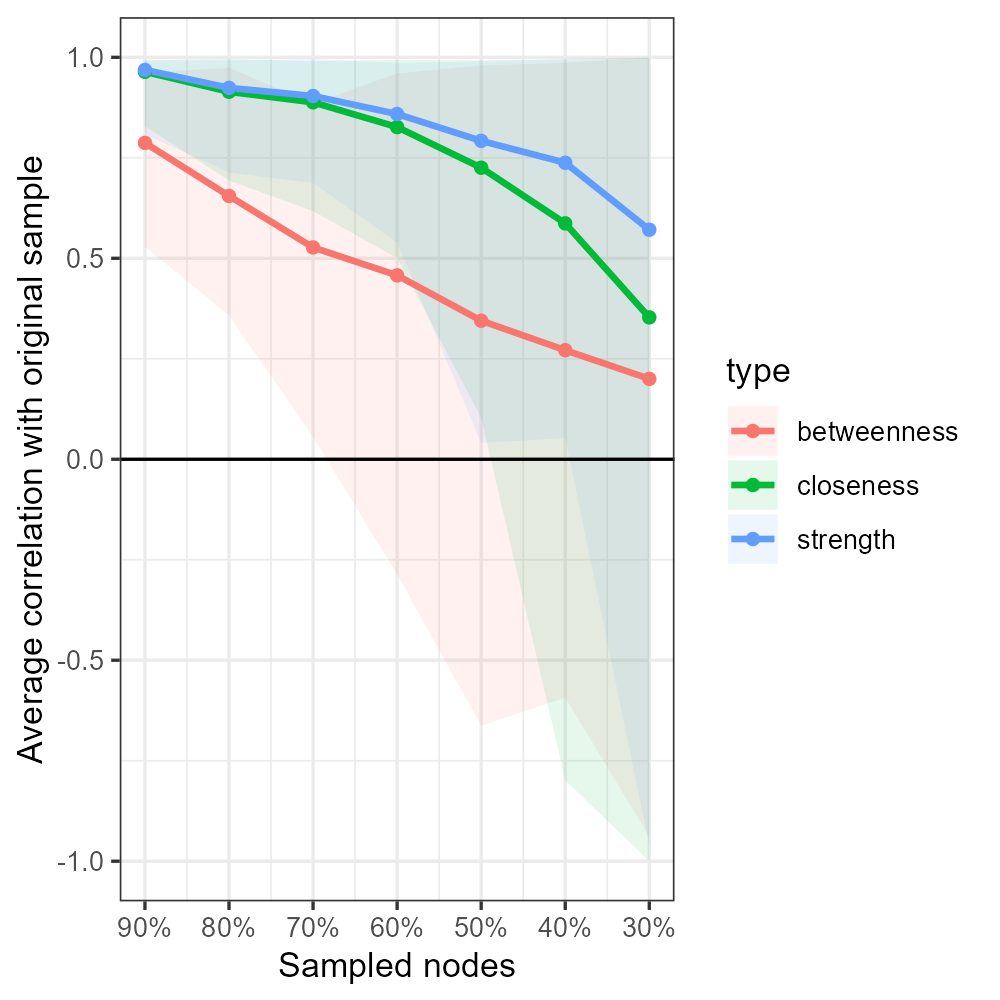


**Supplementary table 1: Weights matrix of the outcomes measures in the network analysis**

Symp= Symptom severity; Funct: Functioning ( SOFAS total score of the last year); Recov=QPR-15 Total Score; SocDis= Social disadvantage score: PhyDis= Physical disadvantage score; Abuse= ASI (Addiction Severity Index ) total score last year; Episod= number of episodes; SuicAtt= number of suicide attempts; DYAps= Dose-years of antipsychotic drugs; Diagn= DSM 5 final diagnosis (three groups); SCIP: Cognitive score ([The screen for cognitive impairment in psychiatry](https://bmcpsychiatry.biomedcentral.com/articles/10.1186/s12888-021-03508-4); Motor= Motor abnormalities;
